# 4-Methyl-3-penten-2-on – Bestimmung von 4-Methyl-3-penten-2-on (Mesityloxid) in der Luft am Arbeitsplatz mittels Hochleistungsflüssigkeits­chromatographie (HPLC-DAD): Luftanalysen-Methode

**DOI:** 10.34865/am14179d10_2or

**Published:** 2025-06-30

**Authors:** Lutz Nitschke, Adela Frenzen, Dietmar Breuer, Ralph Hebisch, Andrea Hartwig, MAK Commission

**Affiliations:** 1 Bayerisches Landesamt für Gesundheit und Lebensmittelsicherheit Pfarrstraße 3 80538 München Deutschland; 2 Hochschule Bonn-Rhein-Sieg. Fachbereich Angewandte Naturwissenschaften Grantham-Allee 20 53757 Sankt Augustin Deutschland; 3 Bundesanstalt für Arbeitsschutz und Arbeitsmedizin (BAuA) Friedrich-Henkel-Weg 1–25 44139 Dortmund Deutschland; 4 Institut für Angewandte Biowissenschaften. Abteilung Lebensmittelchemie und Toxikologie. Karlsruher Institut für Technologie (KIT) Adenauerring 20a, Geb. 50.41 76131 Karlsruhe Deutschland; 5 Ständige Senatskommission zur Prüfung gesundheitsschädlicher Arbeitsstoffe. Deutsche Forschungsgemeinschaft, Kennedyallee 40, 53175 Bonn, Deutschland. Weitere Informationen: Ständige Senatskommission zur Prüfung gesundheitsschädlicher Arbeitsstoffe | DFG

**Keywords:** 4-Methyl-3-penten-2-on, Luftanalysen, Analysenmethode, Arbeitsplatzmessung, Gefahrstoff, Hochleistungsfüssigkeits­chromatographie, Diodenarray-Detektion, HPLC-DAD, Silicagel, Flüssigdesorption, 4-methyl-3-penten-2-one, air analyses, analytical method, workplace measurement, hazardous substance, high-performance liquid chromatography, diode array detection, HPLC-DAD, silica gel, liquid desorption

## Abstract

The working group “Air Analyses” of the German Senate Commission for the Investigation of Health Hazards of Chemical Compounds in the Work Area (MAK Commission) developed and verified the presented analytical method. It is used to determine the levels of 4-methyl-3-penten-2-one [141-79-7] that occur in the workplace air. The method covers concentrations in the range from one tenth up to twice the current occupational exposure limit value (OELV) of 8.1 mg/m^3^. The method is also suitable for verifying the short-term exposure limit (STEL; excursion factor 2). Samples are collected by drawing a defined volume of air through a sampling tube filled with silica gel using a flow regulated pump at a volumetric flow rate of 0.5 l/min. Exposure during the shift is measured with a sampling period of 2 hours and the short-term exposure with a period of 15 minutes. The 4-methyl-3-penten-2-one adsorbed to the silica gel is extracted by liquid extraction with methanol and analysed by high-performance liquid chromatography using diode array detection. The quantitative determination is based on multiple-point calibrations with external standards. A relative limit of quantification (LOQ) of 0.06 mg/m^3^ is obtained for an air sample volume of 60 litres. As the LOQ for a sample volume of 30 litres is 0.03 mg/m^3^, the STEL can also be measured. The recovery is approx. 100% and the expanded uncertainty is 14% for a sampling period of 2 hours and below 16% for a period of 15 minutes.

**Table:** 

**Methodennummer**	1
**Anwendbarkeit**	Luftanalyse
**Analyt. Messprinzip**	Hochleistungsflüssigkeitschromatographie mit Diodenarray-Detektion (HPLC-DAD)

## 1 Kenndaten des Verfahrens

**Table:** 

**Präzision:**	Standardabweichung (rel.):	*s* = 1,1–1,4 %
	Erweiterte Messunsicherheit:	*U* = 14 %
	in einem Bereich von 0,81–16,2 mg/m^3^ und n = 6	
**Bestimmungsgrenze:**	3,5 µg absolut pro Probenträger	
	0,06 mg/m^3^ bei einem Probeluftvolumen von 60 l und einer Probenahmedauer von 2 h
**Wiederfindung:**	*η* = 100,4–101,4 %
**Probenahmeempfehlung:**	Probenahmedauer:	2 h
	Probeluftvolumen:	60 l
	Volumenstrom:	0,5 l/min
	Für Kurzzeitmessungen:	15 min; 2 l/min

## 2 Stoffbeschreibung

### 4-Methyl-3-penten-2-on [141-79-7]

**Abb. 1:**
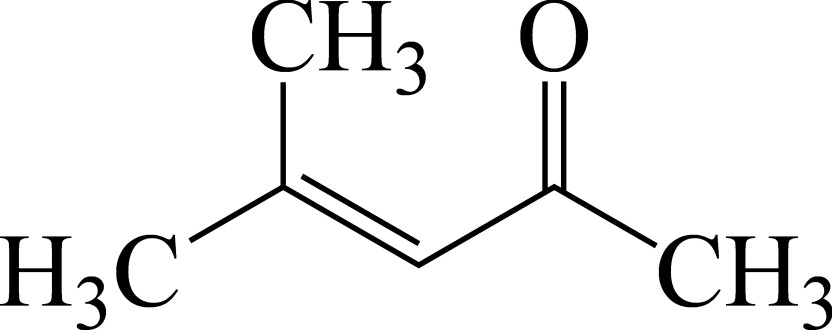
Strukturformel von 4-Methyl-3-penten-2-on

4-Methyl-3-penten-2-on (siehe Abbildung 1, auch Mesityloxid, Isopropylidenaceton, 1-Isobutenylmethylketon, Methylisobutenylketon genannt) ist eine farblose bis gelbliche, ölige Flüssigkeit mit pfefferminzartigem Geruch. Es wird industriell durch Dehydratisierung von Diacetonalkohol gewonnen, der wiederum in einer Aldolreaktion aus Aceton hergestellt wird (IFA 2024; RÖMPP-Redaktion und Jahn 2019). Technisches Mesityloxid kann bis zu 10 % des Isomers 4-Methyl-4-penten-2-on [3744-02-3] enthalten.

Ein Großteil des 4-Methyl-3-penten-2-on wird zu 4-Methylpentan-2-on [108-10-1] (Methylisobutylketon, MIBK) weiterverarbeitet, das in der Lackindustrie als Lösungsmittel für Natur- und Kunstharze eingesetzt wird. 4-Methyl-3-penten-2-on dient weiterhin als Ausgangsstoff für die Synthese bestimmter Terpene und Riechstoffe (RÖMPP-Redaktion und Jahn 2019; RÖMPP-Redaktion 2002).

Der Arbeitsplatzgrenzwert (AGW) und der MAK-Wert betragen 8,1 mg/m^3^ (2 ml/m^3^) in der Luft am Arbeitsplatz. Der Kurzzeitwert ist der Spitzenbegrenzungs-Kategorie I mit dem Überschreitungsfaktor 2 zugeordnet und der Stoff ist mit „H“ (kann in toxikologisch relevanten Mengen über die Haut aufgenommen werden) markiert (AGS 2024; DFG 2024). Stoffdaten zu 4-Methylpent-3-en-2-on können Tabelle 1 entnommen werden.

**Tab. 1:** Stoffdaten zu 4-Methylpent-3-en-2-on (IFA 2024)

Name	4-Methyl-3-penten-2-on
CAS-Nr.	141-79-7
Molmasse [g/mol]	98,14
Aggregatzustand bei 20 °C	flüssig
Dichte bei 25 °C [g/cm^3^]	0,858^a)^
Dampfdruck bei 20 °C [hPa]	10,9
Schmelzpunkt [°C]	–59
Siedepunkt bei 1013 hPa [°C]	130
Flammpunkt [°C]	24
Beurteilungsmaßstäbe	
Deutschland: AGW, MAK-Wert (AGS 2024; DFG 2024)	8,1 mg/m^3^
Spitzenbegrenzungs-Kategorie (Überschreitungsfaktor) (AGS 2024; DFG 2024)	I(2)

^a)^ verwendete Dichte ist dem Sicherheitsdatenblatt des Herstellers entnommen (Sigma-Aldrich 2019)

## 3 Grundlage des Verfahrens

Das Analysenverfahren ermöglicht die Bestimmung des Gehaltes an 4-Methyl-3-penten-2-on in der Luft am Arbeitsplatz im Bereich von einem Zehntel bis zum Doppelten des derzeit gültigen AGW bzw. MAK-Wertes von 8,1 mg/m^3^ (AGS 2024; DFG 2024). Auch die Einhaltung des Kurzzeitwerts mit einem Überschreitungsfaktor von 2 kann überprüft werden (DIN 2021 a). Das Verfahren erfüllt die Anforderungen der TRGS 402 (AGS 2023) in vollem Umfang.

Zur Probennahme wird ein definiertes Luftvolumen mit einer geeigneten Probenahmepumpe durch ein Sorptionsröhrchen (Silicagel) gesaugt. Der Inhalt des beaufschlagten Silicagelröhrchens wird in ein Braunglasgefäß überführt, mit Methanol überschichtet und geschüttelt. Die Bestimmung im Methanolextrakt wird mittels Hochleistungsflüssigkeitschromatographie (HPLC) mit Diodenarray‐Detektion (DAD) durchgeführt. Die quantitative Auswertung erfolgt anhand einer Kalibriergeraden mit externen Standards.

## 4 Geräte, Chemikalien und Lösungen

### 4.1 Geräte

Für die Probenahme:

- Probenahmepumpe für personengetragene und stationäre Probenahme, geeignet für einen Volumenstrom von 0,5 l/min (z. B. SG5200, Fa. GSA Messgerätebau GmbH, 40880 Ratingen)
- Silicagelröhrchen, aktiviertes Silicagel (45/60) 300/150 mg, 8 × 75 mm (z. B. ORBO 506, Supelco, Fa. Merck KGaA, 64271 Darmstadt); ggf. bei hoher Luftfeuchte Silicagelröhrchen größerer Kapazität, Typ B/G, gereinigtes Silicagel, 1100/480 mg, 7 × 122 mm (z. B. Typ B/G, Fa. Drägerwerk AG & Co. KGaA, 23558 Lübeck)
- Durchflussmesser (z. B. Bios DryCal Definer 220, Fa. MesaLabs, vertrieben durch Brandt Instruments, Inc., Prairieville, LA, USA)
- Siliconschlauch mit passender Dimension zur Verbindung von Probenahmepumpe und Silicaröhrchen
- Hygrometer zur Bestimmung der Luftfeuchte

Für die Probenaufbereitung und analytische Bestimmung:

- Reinstwasseranlage zur Herstellung von Reinstwasser (z. B. Millipore-Q-Integral 3, Fa. Merck KGaA, 64271 Darmstadt)
- Variable, direktverdrängende Kolbenhubpipetten, 10–100 µl und 100–1000 µl (z. B. Fa. Gilson, Inc., Middleton, WI, USA)
- Pipettenspitzen (z. B. Fa. Gilson, Inc., Middleton, WI, USA)
- Flaschenaufsatz-Dispenser, 10–50 ml (z. B. RotiLabo-Dispenser, Fa. Carl Roth GmbH + Co. KG, 76185 Karlsruhe)
- Braunglasgefäße, ca. 22 ml, mit Schraubdeckel (z. B. Supelco, Fa. Merck KGaA, 64271 Darmstadt)
- Labor-Schüttler (z. B. IKA-VIBRAX-VBA, Fa. Jahnke & Kunkel GmbH & Co. KG, 79219 Staufen)
- Messkolben aus Glas, 10 ml, mit Glasstopfen (z. B. Fa. BRAND GmbH + Co KG, 97877 Wertheim)
- Einmalspritzen, 10 ml, mit Luer-Lock-Anschluss (z. B. Fa. Henke-Sass, Wolf GmbH, 78532 Tuttlingen)
- Spritzenvorsatzfilter, regenerierte Cellulose, Porengröße 0,45 µm, Ø 25 mm, mit Luer-Lock-Anschluss (z. B. Chromafil RC-45/25, Fa. Macherey-Nagel GmbH & Co. KG, 52355 Düren)
- Hochleistungsflüssigkeitschromatograph, Probengeber mit Kühlfunktion, Säulenofen und DAD (z. B. Dionex UltiMate 3000, Fa. ThermoFisher Scientific GmbH, 633303 Dreieich)
- Autosamplergläschen, 1,5 ml, 11,6 × 32 mm, Braunglas mit Schraubgewinde und Deckel (z. B. Fa. Macherey-Nagel GmbH & Co. KG, 52355 Düren)
- Trennsäule, C18, Länge (L) 250 mm; Innendurchmesser (ID) 4,6 mm; Partikelgröße: 5 μm mit Vorsäule L 20 mm, ID 4 mm, Partikelgröße 5 μm (z. B. Supelcosil LC‑18 mit Supelguard LC-18, Fa. Merck KGaA, 64271 Darmstadt)
- Röhrchenöffner (z. B. TO 7000, Fa. Drägerwerk AG & Co. KGaA, 23558 Lübeck)

### 4.2 Chemikalien

- 4-Methyl-3-penten-2-on, zertifiziertes Referenzmaterial (z. B. PHR1547, Sigma-Aldrich, Fa. Merck KGaA, 64271 Darmstadt)
- Methanol für HPLC, ≥ 99,9 % (z. B. Merck KGaA, 64271 Darmstadt)
- Acetonitril für HPLC, ≥ 99,95 % (z. B. Rotisolv HPLC Ultra Gradient Grade, Fa. Carl Roth GmbH + Co. KG, 76185 Karlsruhe)
- Reinstwasser (ρ ≥ 18,2 MΩ × cm bei 25 °C)

### 4.3 Lösungen 

Unter Verwendung der in Abschnitt 4.2 aufgeführten Chemikalien werden folgende im Kühlschrank bei 4–8 °C über mindestens 3 Monate haltbare Lösungen hergestellt:

**Stammlösung:** (48,6 mg/ml in Methanol)

486 mg des 4-Methyl-3-penten-2-on-Referenzmaterials (566 µl, ρ = 0,858 g/cm^3^) werden in einen mit ca. 2 ml Methanol vorgelegten 10-ml-Messkolben überführt, dieser bis zur Marke mit Methanol aufgefüllt und geschüttelt.

Durch Verdünnungen der Stammlösung werden Arbeitslösungen hergestellt.

**Arbeitslösung 1 (Dotierlösung 1):** 1:5-Verdünnung der Stammlösung (9,72 mg/ml in Methanol)

In einem 10-ml-Messkolben werden ca. 2 ml Methanol vorgelegt. Anschließend werden 2 ml der Stammlösung hinzugegeben, der Kolben mit Methanol auf 10 ml aufgefüllt und geschüttelt.

**Arbeitslösung 2 (Dotierlösung 2):** 1:10-Verdünnung der Stammlösung (4,86 mg/ml in Methanol)

In einem 10-ml-Messkolben werden ca. 2 ml Methanol vorgelegt. Anschließend wird 1 ml der Stammlösung hinzugegeben, der Kolben mit Methanol auf 10 ml aufgefüllt und geschüttelt.

**Arbeitslösung 3 (Dotierlösung 3)**: 1:100-Verdünnung der Stammlösung (486 µg/ml in Methanol)

In einem 10-ml-Messkolben werden ca. 2 ml Methanol vorgelegt. Anschließend werden 0,1 ml der Stammlösung hinzugegeben, der Kolben mit Methanol auf 10 ml aufgefüllt und geschüttelt.

**Kalibrierlösungen:** (Kalibrierlösung 1: 97,2 µg/ml, Kalibrierlösung 2: 48,6 µg/ml, Kalibrierlösung 3: 4,81 µg/ml in Methanol)

Aus den Arbeitslösungen 1, 2 und 3 werden durch 1:100-Verdünnungen mit Methanol die Kalibrierlösungen 1, 2 und 3 hergestellt. Dazu werden in einen 10-ml-Messkolben, in dem ca. 2 ml Methanol vorgelegt wurden, jeweils 0,1 ml der entsprechenden Arbeitslösung überführt, die Kolben bis zur Marke mit Methanol aufgefüllt und geschüttelt.

### 4.4 Kalibrierstandards

Die Kalibrierstandards werden durch Verdünnungen der Kalibrierlösungen mit Methanol entsprechend dem in Tabelle 2 aufgeführten Schema hergestellt. Dazu werden die aufgeführten Volumina der Kalibrierlösungen und des Lösungsmittels Methanol mittels Kolbenhubpipetten in 1,5-ml-Gläschen gefüllt.

**Tab. 2:** Pipettierschema zur Herstellung der Kalibrierstandards und resultierende Konzentrationen

**Kalibrierstandard**	**Kalibrierlösung**	**Konzentration Kalibrierlösung** **[µg/ml]**	**Volumen Kalibrierlösung** **[µl]**	**Volumen Methanol** **[µl]**	**Konzentration Kalibrierstandard** **[µg/ml]**	**Masse pro 10 µl Injektion** **[ng]**
I	3	4,86	5	995	0,0243	0,243
II	3	4,86	10	990	0,0486	0,486
III	3	4,86	20	980	0,0972	0,972
IV	3	4,86	25	975	0,122	1,22
V	3	4,86	50	950	0,243	2,43
VI	3	4,86	100	900	0,486	4,86
VII	3	4,86	200	800	0,972	9,72
VIII	3	4,86	400	600	1,94	19,4
IX	3	4,86	1000	0	4,86	48,6
X	1	97,2	100	900	9,72	97,2
XI	2	48,6	333	667	14,6	146
XII	1	97,2	200	800	19,4	194
XIII	2	48,6	500	500	24,3	243
XIV	2	48,6	600	400	29,2	292
XV	2	48,6	700	300	34,0	340
XVI	2	48,6	800	200	38,9	389
XVII	2	48,6	900	100	43,7	437
XVIII	2	48,6	1000	0	48,6	486
XIX	1	97,2	667	333	64,8	648
XX	1	97,2	833	167	81,0	810
XXI	1	97,2	1000	0	97,2	972
XXII	Dotierlsg. 2	4860	25	975	122	1220

### 4.5 Kontrolllösungen

Kontrolllösungen zur Qualitätskontrolle werden durch Verdünnung (1:100) der Arbeitslösungen 1, 2 oder 3 hergestellt. Dazu werden in Braunglasgefäßen mittels Dispenser 10 ml Methanol vorgelegt. Davon werden 100 µl abpipettiert und verworfen. Jeweils 100 µl der Arbeitslösungen 1, 2 oder 3 werden jeweils in das Braunglasgefäß gegeben. Die Kontrolllösung wird arbeitstäglich frisch angesetzt und unter den in Abschnitt 6 genannten Bedingungen analysiert.

## 5 Probenahme und Probenaufbereitung

### 5.1 Probenahme

Die Probenahme kann sowohl ortsfest als auch personengetragen erfolgen. Bei personengetragener Probenahme erfolgt diese im Atembereich. Direkt vor der Probenahme werden die Silicagelröhrchen mit einem Glasschneider geöffnet und über einen Verbindungsschlauch mit der Probenahmepumpe verbunden.

Für die Ermittlung des Schichtmittelwertes wird die Probeluft mit Hilfe der durchflussstabilisierten Pumpe mit einem Volumenstrom von 0,5 l/min für einen Zeitraum von mindestens zwei Stunden durch das Silicagelröhrchen gesaugt. Bei zwei Stunden Probenahme entspricht dies einem Probeluftvolumen von 60 l. Für die Ermittlung des Kurzzeitwertes wird die Luftprobe mit einem Volumenstrom von 2 l/min für einen Zeitraum von 15 Minuten durch das Silicagelröhrchen gesaugt (30 l Probenahmevolumen).

Die für die Bestimmung der Luftkonzentration wichtigen Parameter (Probeluftvolumen, Temperatur und relative Luftfeuchte) werden im Probenahmeprotokoll vermerkt. Insbesondere die relative Luftfeuchte ist bei der Auswahl des geeigneten Probenahmesystems zu berücksichtigen (siehe Abschnitt 10.2).

Nach der Probenahme ist der Volumenstrom auf Konstanz zu überprüfen. Ist die Abweichung vom eingestellten Volumenstrom ≥ ± 5 %, wird empfohlen, die Messung zu wiederholen (DIN 2023).

### 5.2 Probenaufbereitung

Aufgrund der ermittelten Lagerfähigkeit (siehe Abschnitt 10.6), müssen die Silicagelröhrchen nach der Probenahme innerhalb von 2 Wochen aufgearbeitet werden. Der gesamte Inhalt jedes Röhrchens (d. h. inklusive der Kontrollschicht und der Glaswolle) wird in jeweils ein Braunglasgefäß überführt, in dem 10 ml Methanol vorgelegt sind. Danach werden die Braunglasgefäße in einen Laborschüttler eingesetzt und 30 Minuten bei 400 bis 500 Umdrehungen pro Minute geschüttelt. Die Extrakte werden danach filtriert und ein Aliquot in jeweils ein 1,5-ml-Autosamplergefäß überführt. Diese werden in einem Autosampler platziert und mittels HPLC analysiert.

Zur Bestimmung des Laborblindwertes („Lab Blank“) wird ein Silicagelröhrchen der jeweils verwendeten Charge mit 100 µl Methanol dotiert und dem gesamten Aufarbeitungsverfahren unterzogen.

## 6 Instrumentelle Arbeitsbedingungen

**Table:** 

**Gerät:**	Hochleistungsflüssigkeitschromatograph mit automatischem Probengeber, Säulenofen und DAD, z. B Dionex Ultimate 3000, ThermoFisher Scientific GmbH
**Vorsäule:**	Supelguard LC-18, ID 4 mm, L 20 mm, Partikelgröße 5 µm
**Trennsäule:**	Supelcosil LC‑18, ID 4,6 mm, L 250 mm, Partikelgröße 5 µm
**Säulentemperatur:**	30 °C
**Detektor:**	Diodenarray-Detektor
**Wellenlänge:**	Scan: 200–550 nm, Quantifizierung: 239 nm
**Mobile Phase:**	Acetonitril/Reinstwasser, 1:1 (V/V), isokratisch
**Flussrate:**	0,6 ml/min
**Injektionsvolumen:**	10 µl
**Laufzeit:**	12 min

Unter den angegebenen Bedingungen hat 4-Methyl-3-penten-2-on eine Retentionszeit von ca. 8,5 Minuten.

## 7 Analytische Bestimmung

Zur analytischen Bestimmung werden jeweils 10 µl der nach Abschnitt 5.2 aufbereiteten Proben in den Hochleistungsflüssigkeitschromatographen injiziert und unter den in Abschnitt 6 angegebenen Bedingungen analysiert. Liegen die ermittelten Konzentrationen oberhalb des Kalibrierbereiches, so sind geeignete Verdünnungen in Methanol herzustellen und diese nochmals zu analysieren.

## 8 Kalibrierung

**Abb. 2:**
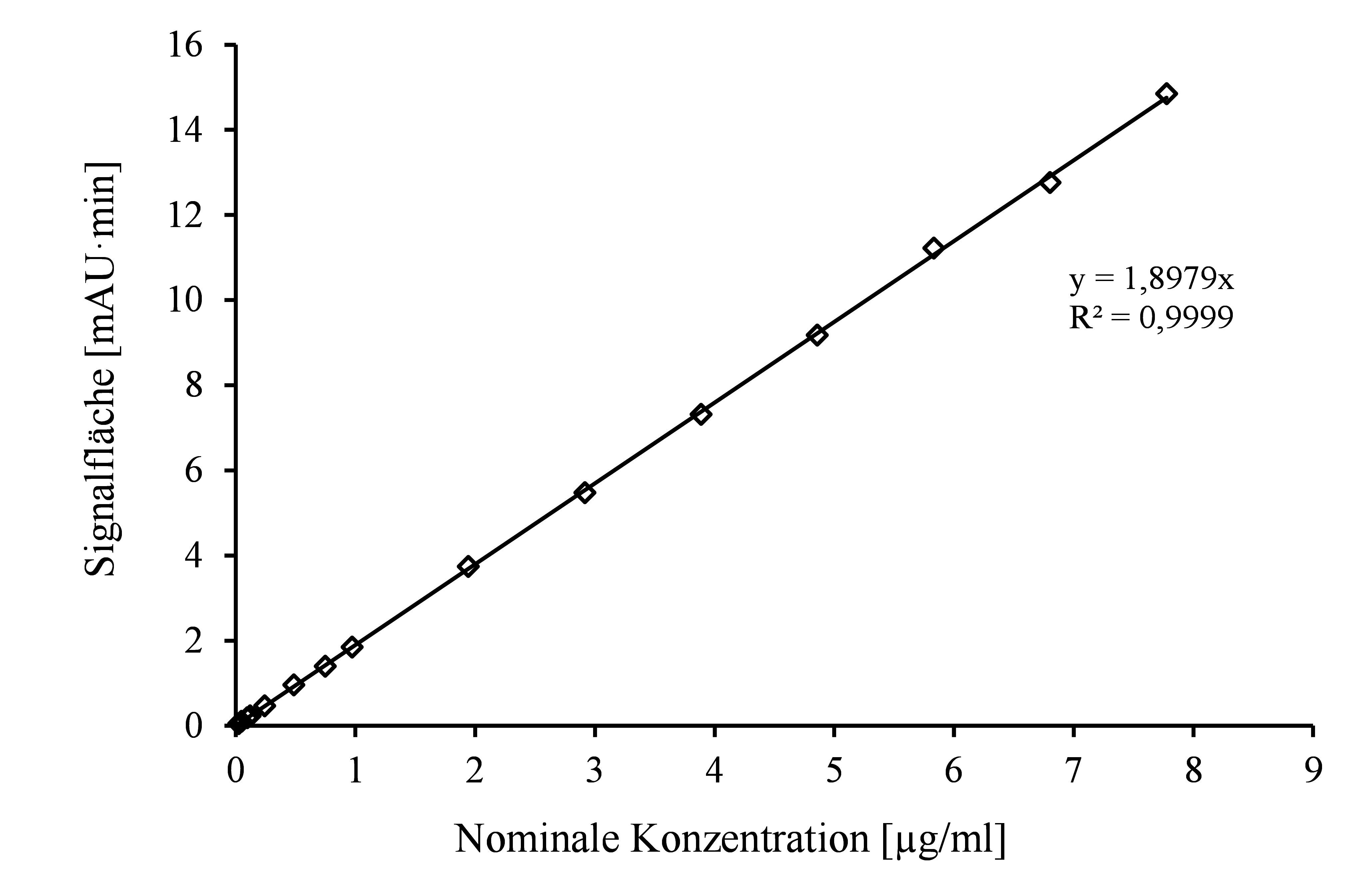
Kalibrierfunktion von 4-Methyl-3-penten-2-on im Konzentrationsbereich der Bestimmungsgrenze

Zur Erstellung der Kalibrierfunktion werden die unter Abschnitt 4.4 beschriebenen Kalibrierstandards entsprechend Abschnitt 6 und 7 analysiert und die ermittelten Peakflächen gegen die jeweiligen Konzentrationen aufgetragen. Die Kalibrierfunktion ist im untersuchten Konzentrationsbereich linear (siehe Abbildung 2).

Zur Überprüfung der Kalibrierfunktion sind arbeitstäglich Qualitätskontrolllösungen (Abschnitt 4.5) zu analysieren. Die Kalibrierung ist neu zu erstellen, wenn die analytischen Bedingungen sich ändern oder die Qualitätskontrolle dazu Anlass gibt.

## 9 Berechnung des Analysenergebnisses

Die Berechnung der Konzentration von 4-Methyl-3-penten-2-on in der Luft am Arbeitsplatz (*ρ*) erfolgt mit Hilfe der von der Datenauswerteeinheit berechneten Konzentration in der Messlösung. Die Datenauswerteeinheit verwendet dazu die erstellte Kalibrierfunktion. Aus der Konzentration in der Messlösung wird unter Berücksichtigung des Desorptionsvolumens, des Blindwertes für 4-Methyl-3-penten-2-on und des Probeluftvolumens die Konzentration an 4-Methyl-3-penten-2-on in der Luft im Arbeitsbereich nach Gleichung 1 berechnet.



(1)

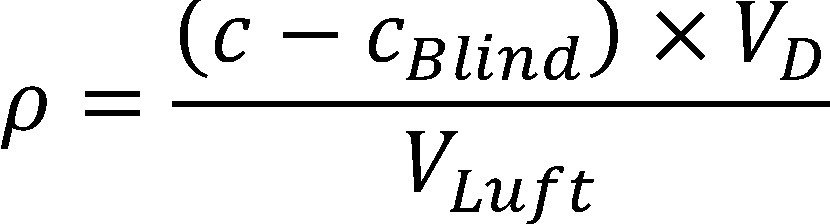




Zur Umrechnung auf 20 °C und 1013 hPa gilt Gleichung 2:



(2)

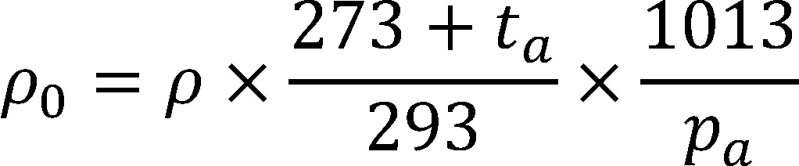




Es bedeuten:

**Table:** 

*ρ*	Massenkonzentration an 4-Methyl-3-penten-2-on in der Luftprobe in mg/m^3^ bezogen auf *t_a_* und *p_a_*
*ρ_0_*	Massenkonzentration an 4-Methyl-3-penten-2-on in mg/m^3^ bezogen auf 20 °C und 1013 hPa
*c*	Konzentration an 4-Methyl-3-penten-2-on in der Messlösung in mg/l
*c_Blind_*	Konzentration des Blindwertes in mg/l
*V_D_*	Volumen der Desorptionslösung in l
*V_Luft_*	Probeluftvolumen in m^3^ (ermittelt aus Volumenstrom und Probenahmedauer, hier 0,06 m^3^)
*t_a_*	Temperatur während der Probenahme in °C
*p_a_*	Luftdruck während der Probenahme in hPa

## 10 Beurteilung des Verfahrens

Die Kenndaten der Methode wurden gemäß DIN EN 482 (DIN 2021 a), DIN EN ISO 22065 (DIN 2021 b) sowie DIN 32645 (DIN 2008) ermittelt. Es wurde eine vollständige Validierung der Methode durchgeführt.

### 10.1 Präzision, Wiederfindung und erweiterte Messunsicherheit

Zur Ermittlung der Präzision und der erweiterten Messunsicherheit wurden jeweils sechs Silicagelröhrchen mit drei unterschiedlichen Massen an 4-Methyl-3-penten-2-on dotiert. Die dotierten Massen wurden dabei so gewählt, dass sie bei einem Probenahmevolumen von 60 l einem Zehntel des AGW, dem AGW und dem Doppelten des AGW entsprechen. Dazu wurden jeweils sechs Silicagelröhrchen mit je 100 µl der Dotierlösung 1, 2 und 3 (siehe Abschnitt 4.3) dotiert. Durch die belegten Silicagelröhrchen wurde anschließend mittels Probenahmepumpe für zwei Stunden Luft mit einem Volumenstrom von 0,5 l/min, einer Temperatur von ca. 25 °C und einer relativen Luftfeuchte von ca. 30 % gesaugt. Anschließend wurden die Sammelröhrchen allen Schritten der Aufbereitung und Analytik, wie unter den Abschnitten 5.2, 6 und 7 beschrieben, unterworfen. Aus den Ergebnissen wurden die Präzisions- und Wiederfindungsdaten ermittelt, die in Tabelle 3 dargestellt sind.

**Tab. 3:** Wiederfindung, relative Standardabweichung und erweiterte Messunsicherheit *U* für n = 6 Bestimmungen

**Dotierte Masse** **[µg]**	**Konzentration^a)^** **[mg/m^3^]**	**Wiederfindung** **[%]**	**Standardabweichung (rel.)** **[%]**	**Erw. Messunsicherheit *U*** **[%]**
48,6	0,81	100,4	1,4	14,2
486	8,1	101,4	1,1	14,3
972	16,2	101,1	1,4	14,3

^a)^ Die Konzentration ergibt sich für eine zweistündige Probenahme bei einem Volumenstrom von 0,5 l/min.

Die erweiterte Messunsicherheit wurde unter Abschätzung aller relevanten Einflussgrößen ermittelt. Die Ergebnisunsicherheit umfasst zwei wesentliche Beiträge, die Unsicherheitskomponenten der Probenahme gemäß Anhang B der DIN EN 482 (DIN 2021 a) und der Analyse.

Parallel wurde zu jeder Dotierreihe eine Blindwertbestimmung (siehe Abschnitt 5.2) durchgeführt.

### 10.2 Einfluss der Luftfeuchte

Der Einfluss der Luftfeuchte auf die Wiederfindung wurde bei Konzentrationen vom 0,1- und 2-Fachen des AGW bei Raumtemperatur (ca. 20–25 °C) und einem Volumenstrom von 0,5 l/min untersucht. Dazu wurden für jede Einzeluntersuchung jeweils vier Silicagelröhrchen mit den Dotierlösungen 1, 2 bzw. 3 belegt und im Anschluss für die angegebene Dauer mittels Probenahmepumpe Luft mit einem Volumenstrom von 0,5 l/min und unterschiedlicher relativer Luftfeuchte durch die Röhrchen gesaugt. Die Ergebnisse der Untersuchungen bei verschiedenen relativen Luftfeuchten, Probenahmezeiten und Dotiermengen sind in Tabelle 4 dargestellt.

**Tab. 4:** Einfluss der relativen Luftfeuchte auf die Wiederfindung von 4-Methyl-3-penten-2-on bei Raumtemperatur

**Rel. Luftfeuchte** **[%]**	**Dotierte Masse** **[µg]**	**Probenahmedauer** **[min]**	**Probeluftvolumen** **[l]**	**AGW**	**Wiederfindung** **[%]**	**Standardabweichung (rel.)** **[%]**
Silicagelröhrchen ORBO 506
20	48,6	120	60	0,1	102,9	0,8
20	972	120	60	2,0	102,0	0,5
30	48,6	120	60	0,1	101,0	0,9
30	972	120	60	2,0	100,3	1,5
50	486	60	30	2,0	98,0	1,2
50	972	120	60	2,0	96,5	1,2
60	24,4	60	30	0,1	101,5	7,6
60	486	60	30	2,0	100,9	4,2
60	48,6	120	60	0,1	63,3	1,4
60	972	120	60	2,0	40,1	11,5
Silicagelröhrchen Dräger Typ B/G
80	24,4	60	30	0,1	97,4	1,6
80	486	60	30	2,0	101,8	1,5
80	48,6	120	60	0,1	95,6	1,6
80	972	120	60	2,0	94,3	2,9

Bei einer relativen Luftfeuchte ab 60 % kommt es zu Verlagerungen des adsorbierten 4-Methyl-3-penten-2-on mit dem Probenahmeluftstrom. Deshalb muss in diesem Fall die Probenahmedauer verkürzt und/oder der Volumenstrom vermindert werden, um das Probenahmevolumen zu reduzieren; ein Luftvolumen von 30 l sollte dabei nicht überschritten werden.

Alternativ können zwei Silicagelröhrchen (Typ ORBO 506) hintereinander angeordnet oder ein Silicagelröhrchen mit größerer Kapazität verwendet werden (z. B. Dräger Typ B/G). Tabelle 4 zeigt die Wiederfindung von 4-Methyl-3-penten-2-on auf Sammelröhrchen des Typs B/G von Dräger bei 80 % relativer Luftfeuchte. Aus den Wiederfindungsversuchen ist erkennbar, dass auch bei Verwendung von Dräger Silicagelröhrchen Typ B/G das Probenahmevolumen bei relativen Luftfeuchten von 80 % nicht größer als 30 Liter sein sollte.

Die Ergebnisse zeigen, dass parallel zur Probenahme immer auch die Luftfeuchte zu ermitteln ist!

### 10.3 Einfluss der Temperatur

Der Einfluss der Temperatur auf die Wiederfindung wurde bei 5 °C und 40 °C geprüft. Die Untersuchungen fanden bei einer relativen Luftfeuchte von ca. 20–25 % statt. Zur Simulation des Zweifachen des AGW wurden zwei Silicagelröhrchen mit jeweils 100 µl der Dotierlösung 1 (9,72 mg/ml, siehe Abschnitt 4.2) dotiert und mittels Probenahmepumpe für zwei Stunden Luft mit einem Volumenstrom von 0,5 l/min durch die Röhrchen gesaugt. Die Ergebnisse der Untersuchungen sind in Tabelle 5 dargestellt.

**Tab. 5:** Einfluss der Temperatur auf die Wiederfindung von 4-Methyl-3-penten-2-on bei einer relativen Luftfeuchte von 20–25 %

**Temperatur** **[°C]**	**Dotierte Masse** **[µg]**	**Probenahmedauer** **[min]**	**Luftvolumen** **[l]**	**AGW**	**Wiederfindung** **[%]**
5	972	120	60	2,0	100,1
40	972	120	60	2,0	102,8

Wie in Tabelle 5 gezeigt ist, hat unter den angegebenen Probenahmebedingungen die Umgebungstemperatur keinen Einfluss auf die Wiederfindung.

Bei Temperaturen bis 40 °C und einstündiger Probenahme hat eine relative Luftfeuchte von bis zu 40 % keinen negativen Einfluss auf das Messergebnis (vgl. Abschnitt 10.2).

### 10.4 Bestimmungsgrenze

Als Grundlage für die Ermittlung der Bestimmungsgrenze dient die Norm DIN 32645 (DIN 2008). Nach Durchführung einer 15-Punkt-Kalibrierung im niedrigen Konzentrationsbereich von 0,0243–7,78 µg/ml und 10 µl Injektionsvolumen wurde die Bestimmungsgrenze berechnet. Diese beträgt 3,5 µg 4-Methyl-3-penten-2-on absolut oder 0,06 mg/m^3^ bezogen auf ein Probeluftvolumen von 60 Litern (0,5 l/min Pumpendurchfluss und 2 h Probenahme).

### 10.5 Kapazität des Probenahmesystems

Zur Bestimmung des Durchbruchverhaltens wurden jeweils zwei Silicagelröhrchen mit einem Siliconschlauchstück verbunden. Das vordere Röhrchen wurde entweder mit 100 µl Dotierlösung 1 für die Überprüfung des Zweifachen des AGW oder mit 200 µl Dotierlösung 1 für die Überprüfung des Vierfachen des AGW belegt. Anschließend wurde bei Raumtemperatur mittels Probenahmepumpe Luft mit einer Flussrate von 0,5 l/min und einer relativen Luftfeuchte von etwa 30 % durch die Röhrchen gesaugt. Nach zwei Stunden simulierter Probenahmedauer konnte kein Durchbruch nachgewiesen werden. Die Wiederfindung lag bei durchschnittlich 102,9 %.

### 10.6 Lagerfähigkeit

Die Lagerfähigkeit der beaufschlagten Silicagelröhrchen wurde nach drei, sieben und 14 Tagen Lagerung bei Raumtemperatur bestimmt. Tabelle 6 zeigt die Wiederfindung als Mittelwerte einer Doppelbestimmung.

**Tab. 6:** Einfluss der Lagerdauer beaufschlagter Silicagelröhrchen auf die Wiederfindung von 4-Methyl-3-penten-2-on bei Raumtemperatur

**AGW**	**Wiederfindung** **3 Tage** **[%]**	**Wiederfindung** **7 Tage** **[%]**	**Wiederfindung** **14 Tage** **[%]**
0,1	104,8	102,5	99,2
2,0	101,3	98,2	96,1

Aus Tabelle 6 ist ersichtlich, dass die Lagerfähigkeit der beaufschlagten Röhrchen von zwei Wochen abgesichert ist.

Zusätzlich wurde die Lagerfähigkeit der Extrakte anhand von sechs unabhängigen Probelösungen untersucht. Die methanolischen Probenextrakte sind im Kühlschrank (ca. 6–8 °C) für einen Monat lagerfähig (siehe Tabelle 7).

**Tab. 7:** Einfluss der Lagerdauer (ca. 6–8 °C) der Extraktionslösungen auf die Wiederfindung

**AGW**	**Wiederfindung** **10 Tage** **[%]**	**Standardabweichung (rel.)** **10 Tage** **[%]**	**Wiederfindung** **1 Monat** **[%]**	**Standardabweichung (rel.)** **1 Monat** **[%]**
0,1	101,1	1,0	102,1	1,5
1,0	101,6	0,5	101,9	0,7
2,0	101,7	0,9	100,3	0,8

### 10.7 Selektivität

Das Analysenverfahren mittels HPLC ist unter den angegebenen Arbeitsbedingungen spezifisch und robust. Es konnten keine Störungen beobachtet werden. Das Isomer 4-Methyl-4-penten-2-on [3744-02-3] wird unter den genannten Arbeitsbedingungen mit der Methode auch erfasst.

## 11 Diskussion

Das hier beschriebene Messverfahren ermöglicht die Bestimmung von 4-Methyl-3-penten-2-on in der Luft am Arbeitsplatz in einem Konzentrationsbereich von einem Zehntel bis zum Doppelten des derzeit gültigen MAK-Wertes bzw. AGW von 8,1 mg/m^3^. Das Messverfahren ist ebenfalls geeignet, die Einhaltung des Kurzzeitwertes zu überprüfen.

Aus den Untersuchungen ist ersichtlich, dass es bei einer relativen Luftfeuchte ab 60 % und einer Probenahmedauer von 120 Minuten zu Verlusten des adsorbierten 4-Methyl-3-penten-2-on mit dem Probenahmeluftstrom kommt. Deshalb muss unter diesen Bedingungen die Probenahmedauer verkürzt und/oder der Volumenstrom vermindert werden. Weitere mögliche Anpassungen der Probenahme bei hohen relativen Luftfeuchten sind im Abschnitt 10.3 beschrieben.

Grundsätzlich sind die Analysebedingungen an das jeweils verwendete HPLC-Messsystem anzupassen.
